# *Chinese Journal of Cancer* reviewer acknowledgement 2015

**DOI:** 10.1186/s40880-016-0090-6

**Published:** 2016-03-18

**Authors:** Rui-Hua Xu, Sarah M. Theissen

**Affiliations:** Department of Medical Oncology, Sun Yat-sen University Cancer Center, 651 Dongfeng Road East, Guangzhou, 510060 Guangdong China; BioMed Central, 236 Gray’s Inn Road, London, WC1X 8HB UK

## Contributing reviewers

The Editors of *Chinese Journal of Cancer* would like to thank all our reviewers who have contributed to the journal in 2015. 

Without the participation of skilful reviewers no academic journal could succeed and we are grateful to the committed individuals who have given their time and expertise to the peer review of manuscripts for *Chinese Journal of Cancer*. We look forward to your continued support in 2016.

**Jin-Xin Bei**

China

**Qing-Qing Cai**

China

**Zheng Cao**

USA

**Sumei Cao**

China

**Xinping Cao**

China

**Ming Chen**

China

**Zhongping Chen**

China

**Hankui Chen**

USA

**Jun Chen**

China

**Hui Chen**

China

**Nianyong Chen**

China

**Hongyan Chen**

China

**Tao Chen**

China

**Ming-Yuan Chen**

China

**Qiong Chen**

China

**GuoQiang Chen**

China

**Jindong Chen**

USA

**Zhe-Sheng Chen**

USA

**Gong Chen**

China

**Xiaodong Cheng**

China

**Shi-Yuan Cheng**

USA

**Zhibin Cheng**

China

**Niall Corcoran**

Canada

**Yiwei Cui**

China

**Bingbing Dai**

USA

**Yun Dai**

USA

**Xin Du**

China

**Chaojun Duan**

China

**Yun Fan**

China

**Wei Fan**

China

**Qipeng Fan**

USA

**Liwu Fu**

China

**Xianshu Gao**

China

**Song Gao**

China

**Guoquan Gao**

China

**Minghua Ge**

China

**Cuizhi Geng**

China

**Dongmin Gu**

USA

**Rongping Guo**

China

**Zhigang Guo**

USA

**Jun Guo**

China

**Mingzhou Guo**

China

**Hui Han**

China

**Xiaohui He**

China

**Biao He**

USA

**Jiang He**

USA

**Qiang He**

China

**Chaosu Hu**

China

**Zheyu Hu**

USA

**Xiaojun Huang**

China

**Zhouguang Hui**

China

**Hongbin Ji**

China

**Wei-Hua Jia**

China

**Lintao Jia**

China

**Tiebang Kang**

China

**Sanath Kumar**

USA

**Xiao Li Lan**

China

**Xiang-Ming Lao**

China

**Nan Li**

USA

**Jiancheng Li**

China

**Zhi-Ming Li**

China

**Yuhong Li**

China

**Xin-Jian Li**

USA

**Guancheng Li**

China

**Jinfeng Li**

China

**Bing Li**

China

**Jingao Li**

China

**Jinjun Li**

China

**Jin Li**

China

**Li-Li Li**

Hong Kong

**Qin Li**

China

**Chao Li**

China

**Hua Li**

China

**Jun Li**

China

**Yanfang Li**

China

**Shengping Li**

China

**Shaojun Lin**

China

**Qing Liu**

China

**Yueping Liu**

China

**Yongyu Liu**

USA

**Ran-Yi Liu**

China

**Yong-Feng Liu**

China

**Li Liu**

China

**Guoyan Liu**

China

**Hao Long**

China

**Herbert Loong**

China

**You Lu**

China

**Shun Lu**

China

**Junhang Luo**

China

**Yue Lv**

China

**Jie Ma**

China

**Jun Ma**

China

**Haiqiang Mai**

China

**Jaap Middeldorp**

The Netherlands

**Koji Mita**

Japan

**Yong-Gao Mou**

China

**Jianyun Nie**

China

**Na Niu**

USA

**Jianji Pan**

China

**Han Peng**

USA

**Ji Peng**

China

**Francesco Pezzella**

UK

**Chao-Nan Qian**

China

**Haide Qin**

USA

**Shuangjian Qiu**

China

**Derek Raghavan**

USA

**Zefang Ren**

China

**Jun Ren**

China

**Maryam Sedghi**

Iran

**Jian-Yong Shao**

China

**Lin Shen**

China

**Kunwei Shen**

China

**Yanxia Shi**

China

**Zhi Shi**

China

**Yuqin Song**

China

**Ming Song**

China

**Tianqiang Song**

China

**Yan Sun**

China

**Min-Han Tan**

Singapore

**Yongguang Tao**

China

**Ling Tian**

China

**Dean Tian**

China

**Jinghai Wan**

China

**Yan Wang**

USA

**Lian-Tang Wang**

China

**Zhiqiang Wang**

China

**Jin Wang**

China

**Jianbing Wang**

China

**Shixuan Wang**

China

**Yongbo Wang**

China

**Yongming Wang**

China

**Zehua Wang**

China

**Yu Wang**

China

**Guiying Wang**

China

**Zhimin Wang**

China

**Xiang Wang**

China

**Joseph Wee**

Singapore

**Wei Wei**

USA

**Xiaohua Wu**

China

**Anhua Wu**

China

**Jiangxue Wu**

China

**Lingying Wu**

China

**Peihong Wu**

China

**Liangping Xia**

China

**Conghua Xie**

China

**Wanghong Xu**

China

**Li Xu**

China

**Bing Xu**

China

**Xundi Xu**

China

**Li Yan**

USA

**Jian Yan**

USA

**Jilong Yang**

China

**Kunyu Yang**

China

**AnKui Yang**

China

**Ziming Yu**

USA

**Sheng-Ji Yu**

China

**Eugene Yu**

Canada

**Ping Yuan**

China

**Yuan Yuan**

China

**Yan Yuan**

USA

**Musheng Zeng**

China

**Jian Zhang**

China

**L. Harris Zhang**

USA

**Li Zhang**

China

**Guo-Jun Zhang**

China

**Lanjun Zhang**

China

**Quan Zhang**

China

**Li Zhang**

China

**Jianing Zhang**

China

**Xiaoshi Zhang**

China

**Wen-Hao Zhang**

China

**Wei Zhang**

USA

**Ni Zhao**

USA

**Xianda Zhao**

USA

**Dinghai Zheng**

USA

**Dinghai Zheng**

USA

**Zhendong Zhong**

USA

**Chong Zhong**

China

**Zhiwei Zhou**

China

**Jingjiao Zhou**

USA

**Xiaoyan Zhou**

China

**Huaijun Zhou**

China

**Jun Zhou**

China

**Fangjian Zhou**

China

**Donghui Zhu**

USA

**Xiaofeng Zhu**

China

**Tao Zhu**

China

**Minghua Zhu**

China

